# Work Disparities and the Health of Nurses in Long-Term Care: A Scoping Review

**DOI:** 10.3390/healthcare12202065

**Published:** 2024-10-17

**Authors:** Lynn Shaw, Mehvish Masood, Kimberly Neufeld, Denise Connelly, Meagan Stanley, Nicole A. Guitar, Anna Garnett, Anahita Nikkhou

**Affiliations:** 1 School of Occupational Therapy, Western University, London, ON N6A 3K7, Canada; 2 School of Physical Therapy, Western University, London, ON N6A 3K7, Canada; mmasoo24@uwo.ca (M.M.); dconnell@uwo.ca (D.C.); nguitar@uwo.ca (N.A.G.); 3 Faculty of Health Sciences, Western University, London, ON N6A 3K7, Canada; kneufel4@uwo.ca; 4 Western Libraries, Western University, London, ON N6A 3K7, Canada; mstanle6@uwo.ca; 5 Arthur Labatt Family School of Nursing, Western University, London, ON N6A 3K7, Canada; agarnet6@uwo.ca; 6 Faculty of Health Sciences, York University, Toronto, ON M3J 1P3, Canada; anahitanikkhou@gmail.com

**Keywords:** work disparities, long term care, continuing care, nursing staff, well-being, health, nursing governance, dissatisfaction, inequality

## Abstract

Work disparities, such as unfairness in pay or unequal distribution of work experienced by nurses in long-term care (LTC), can impact the retention and health of this workforce. **Background**: Despite the significant impact of disparities on nurses’ health in LTC, a literature review on work disparities of nurses in LTC has not been conducted. **Method**: This scoping review aimed to explore the nature and extent of research on meso-level work disparities experienced by nurses in LTC and its links with nurse health and well-being. Five databases were searched: MEDLINE (Ovid), EMBASE (Ovid), PsycINFO (Ovid), SCOPUS, and CINAHL (EBSCO host). **Results**: Of the 5652 articles retrieved, 16 studies (14 quantitative and 2 qualitative) published between 1997 and 2024 met the inclusion criteria. A total of 53 work disparities were identified. Only four articles investigated the association of a work disparity with a variable of health (e.g., physical, mental, or poor general health). **Conclusions**: The results suggest that more attention to how disparities impact nurses’ health and lived experiences is warranted. Meso-level disparities from this review provide an initial basis to consider possibilities in the workplace, especially in supporting equity and opportunities for health and well-being at work (e.g., through fair access to professional growth opportunities and a more equitable balance of work expectations and demands of nursing staff). Future studies of the intersection of macro- and meso-level factors are needed to inform better workplace practices and social and economic policies to support the well-being, health, and safety of nurses at work in LTC.

## 1. Introduction

Maintaining adequate workforce participation and retention of nurses in the long-term care (LTC) sector is a global issue of interest [1,2,3]. A higher demand for LTC services [4] is expected due to the rapidly growing aging population [5]. Further, the LTC sector has experienced challenges in recruiting and retaining nurses, with projected nursing staffing shortages estimated to be approximately 58,600 by 2029 in Canada and 63,720 by 2030 in the United States [4,6]. Supporting the health and well-being of nurses and healthcare workers will be essential to respond to these demographic changes and retention demands. Galanis et al. [7] conducted a cross-sectional study on healthcare workers in any clinical setting in Greece and measured job burnout and job satisfaction following the COVID-19 pandemic (in June 2023). Some of their key findings [7] were that nurses experienced high levels of job burnout (91.1%) and low levels of job satisfaction (61.0%). Further, being in the nursing profession was associated with job burnout and job satisfaction, with nurses experiencing greater job burnout and lesser job satisfaction in comparison to other types of healthcare workers [7]. Within another recent scoping review on the experiences of nurses and other healthcare workers in long-term care during the COVID-19 pandemic, Boamah et al. [8] identified three themes related to nurses’ and other healthcare workers’ health and well-being during the pandemic: (1) carrying the load (i.e., the emotional load and distress associated with caring for residents who were suffering from COVID-19); (2) building pressure and burning out (i.e., the symptoms of burnout and mental health issues experienced by nurses and other healthcare workers); and (3) working through it (i.e., the impacts of fear of contagion and feelings of lacking support and recognition during the COVID-19 pandemic). In addition to these negative, the scoping review also found that some studies identified positive impacts on nurses and other healthcare workers—specifically, “positive feelings…including a sense of duty and commitment to care and gratification and fulfillment working during the crisis and how the work contributed to the greater good of society” (p. 1129) [8]. Authors of the review [8] proposed the creation of safe and healthy work environments, a provision of appropriate recognition, and the development of creative and innovative programs as possible ways to promote and support the health and well-being of nurses and other healthcare workers in long-term care.

Systemic inequities and social challenges facing the LTC sector can lead to unintentional work disparities [9], thereby impacting the well-being of workers. For instance, a common work disparity experienced by nurses is the difference in pay within the work sector [10]. When filling shifts during the COVID-19 pandemic, higher-paid agency external nurses were required, as the few remaining lower-paid in-house nurses were already performing double shifts and managing heavy workloads [11]. Other disparities can be related to experiences of mismatch between demands and job resources in the workplace [12], including increased mandatory overtime [9] and the unfair distribution of work expected by some workers over others [13,14]. These types of disparities among groups of nurses in LTC are also linked with outcomes of job dissatisfaction [15], along with increased absences from work due to burnout-related strain and stress related to excessive physical or emotional labor [8]. While these examples represent some types of work disparities, the extent of work disparities experienced among groups of nurses in LTC, especially those that negatively impact health and well-being at work, is not fully understood. Further investigation is warranted to inform actions and initiatives to support nurse well-being and retention in LTC.

Work disparity is a complex construct expressed in various ways [16]. For instance, the National Institute of Occupational Safety and Health (NIOSH) [17] utilizes “health inequities” interchangeably with “health disparities” when drawing upon medical and social determinants of health models. Ndugga et al. [18] described disparities as “differences in health and healthcare between groups that stem from broader social and economic inequities”. Other macro-level factors that are important for understanding how work disparities are shaped were noted in the conceptual framework by Shaw et al. [16]. These included work and employment policies; shifting societal or healthcare norms on work expectations and demands; policies related to work conditions, human rights, safety legislation, and professional regulations; and contextual issues (e.g., increased demand for LTC spaces with an increased aging population [5] and workforce demands in LTC [1,2,3]). Common across all the papers discussing macro-level factors that contribute to work disparities [16,17,18] is that an intersectional approach is needed to learn more about how disparities are manifested, shaped, and experienced as a result of these factors in different work contexts or sectors. Ultimately, intersectional research will inform both the meso- (workplace level) and macro- (system level) [19] categories of work disparities among groups of workers and has the potential to advance this knowledge within the context of LTC.

Further, recognizing that disparities were inconsistently named or identified, members of the present research team [16] analyzed a sample of the literature to develop a conceptual framework prior to the analysis associated with the present review. According to this framework [16], work disparities are defined as “[i] nequalities or differences experienced between groups of workers or collectives in the following four categories related to work: job security, work compensation, work opportunities, and workplace treatment. These inequalities or differences may be linked to differences in groups of workers and/or in group identities (collectives)”. To initiate these types of analyses, the present scoping review study was designed using the meso-level disparities reflected in the work disparity conceptual framework [16].

Other gaps in knowledge relate to how work disparities among nurses in LTC are associated with health and well-being. For the purposes of this review, health and well-being refer to the overall physical, mental, and social state of individuals and groups achieved through doing the things they need, want, and are expected to do with respect to everyday activities that occupy time and bring meaning and purpose to life. This definition drew on two sources [20,21] and was intended to reflect an occupational perspective on the health and well-being of nurses. Information on links between work disparities to health and well-being may lend insights into ways to improve work practices that guide the day-to-day work of nurses, worker health programs at work, worker benefits, and the retention of nurses in LTC [16]. Le [22] noted that there is a need for health surveillance programs to support further knowledge on how health is impacted by groups of workers experiencing work disparities. Thus, the objectives of this scoping review study were to explore the nature and extent of literature on work disparities among nurses in LTC, with a focus on meso-level disparities and their association with nurse health and well-being. By conducting this review, this analysis is intended to support the identification of gaps in the research literature and opportunities to study work disparities experienced by nurses in LTC.

## 2. Materials and Methods

### 2.1. Study Design

In alignment with Arksey and O’Malley’s proposed framework [23], the present scoping review was conducted via the following stages: (1) identify the research question (a review of literature and discussion of team members supported the identification of the research question); (2) identify the relevant literature (the research team discussed with the team librarian and search terms were defined and revised after an initial search in Medline OVID); (3) study selection (inclusion and exclusion criteria were developed for title and abstract, and full-text screening using Covidence); (4) data extraction (demographic data and data on work disparities and links to health were extracted into a Microsoft Excel file); and (5) data analysis (descriptive summaries using tabular data were completed). The scoping review methodology was selected based on the need to use a systematic process to identify, organize, and analyze information on studies related to disparities experienced by nurses in LTC. The work disparity conceptual framework proposed by Shaw et al. [16], including the definition of work disparities, terminology, and categories presented, was used to guide this review (see Appendix A for definitions of terminology used within the conceptual framework). Covidence software (©2024) [24] was used for the de-duplication and screening processes. We used the Preferred Reporting Items for Systematic Reviews and Meta-Analyses Extension for Scoping Reviews (PRISMA-ScR) Checklist in reporting this study [25] (see Table S1).

### 2.2. Search Strategy

The search strategy was developed and refined in consultation with a Teaching and Learning Librarian who was part of the research team (MS). Two searches were conducted. An initial search was performed on MEDLINE (Ovid), EMBASE (Ovid), PsycINFO (Ovid), CINAHL (EBSCO host), and SCOPUS on 25 January 2024. To update the search and ensure that no additional articles met the inclusion criteria, a subsequent search was conducted on 14 August 2024 in MEDLINE (Ovid), EMBASE (Ovid), PsycINFO (Ovid), and on 27 August 2024 in CINAHL and SCOPUS. No date limits or language limits were applied to the search results. The full search strategy for MEDLINE (Ovid), which was adapted to the requirements of other databases (e.g., SCOPUS’ use of keywords only), can be found in Table S2. RIS files obtained from each database were uploaded to Covidence for further screening.

### 2.3. Study Selection

To facilitate study selection, the following categories provided by the Joanna Briggs Institute (JBI) [26] were used to organize the inclusion and exclusion criteria: (1) phenomenon of interest; (2) context; (3) types of participants; and (4) types of studies.

#### 2.3.1. Phenomenon of Interest

This scoping review included studies that investigated work disparities of nurses in LTC (i.e., nurses in LTC were required to be part of at least one comparator group of an identified work disparity; see Appendix A for the definition of ‘comparator group’). The rationale behind conducting the study was also required due to concerns related to nurse health and well-being. For the purposes of the present analysis, categories of concerns related to health and well-being were grouped into four categories: (1) emotional health (e.g., burnout, stressors related to the emotional strain of caregiving); (2) physical health (e.g., musculoskeletal, sprains, injuries, fatigue, physical strains due to exposures at work); (3) mental health (e.g., conditions listed on IDC-9 codes [27] including depression and anxiety, along with psychological safety factors [28]); and (4) general health (i.e., a non-specific category where health was referred to in general).

#### 2.3.2. Context

Studies that met the inclusion criteria included LTC settings (e.g., nursing homes, personal care homes, and residential care facilities). LTC settings are “residential homes that provide ongoing care to patients whose care needs cannot be met in the community”, where patients predominantly require this care due to “advancing age, disability, or declining health” [29]. Non-residential settings that provided care to patients over a short period of time (e.g., acute care settings, hospitals, and skilled nursing facilities) were excluded from the present analysis.

#### 2.3.3. Types of Participants

Studies that met the inclusion criteria were required to have participants who were regulated and/or licensed nurses (e.g., registered nurses [RNs], registered practical nurses [RPNs], nurse practitioners [NPs], licensed practical nurses [LPNs], enrolled nurses [ENs], and assistant practitioner Nurses [APs]). Studies with an exclusive focus on nursing managers/administrators, nursing students, personal support workers (e.g., nursing aides, healthcare aides, personal care aides, and certified nursing assistants), and non-nursing healthcare personnel were excluded. Further, articles with ambiguity about the nurse’s involvement or where nurses were not clearly categorized as working in LTC settings were excluded.

#### 2.3.4. Types of Studies

Peer-reviewed qualitative, quantitative, and mixed qualitative and quantitative primary studies that were available in English were included in the present analysis. Opinion-based pieces (e.g., editorials), scoping/systematic reviews, dissertations, and gray literature (e.g., newspaper articles and government documents) were excluded. No restrictions on publication year or geographic location were implemented.

### 2.4. Study Screening

Covidence software [24] was used to remove duplicates and conduct screening. Two independent authors (M.M. and A.N. for articles retrieved from the initial search; L.S. and K.N. for articles retrieved from the subsequent search) conducted title and abstract screening on de-duplicated studies identified from the database search. In cases where there were conflicts in the screening, the key inclusion criteria were used to come to a consensus. This included prioritizing the necessity of the study to be informed by concerns related to nurses’ health and wellness, along with work disparity criteria (e.g., the identification of two or more comparator groups as a necessary element to be a work disparity). Through this iterative process, the team came to a consensus in selecting studies for inclusion. Full-text articles were retrieved for studies that meet the inclusion criteria and for studies whose relevance is unclear from the abstract. Two out of three authors (L.S., M.M., and K.N.) then screened each full-text article independently. Disagreements were resolved through discussion to reach a consensus with all full-text reviewers (L.S., M.M., and K.N.).

### 2.5. Data Extraction

Three authors (LS., M.M., and K.N.) extracted and organized data from the included studies using a data extraction form. The development of the data extraction form was informed by the work disparity conceptual framework [16] to support the organization of data related to work disparity variables and comparator groups. In accordance with this framework, work disparities extracted were categorized into meso-level “variable of disparity” categories (i.e., job security, work compensation, work treatment, and work opportunity), comparator group identities, and comparator group subdivision categories. Data were independently extracted and categorized, as applicable, by one author and reviewed by at least one other author for completion (L.S., M.M., and K.N.).

The following demographic data were extracted: author(s), year of publication, country, journal scope, type of study, study aims, methodology, study population, data analysis, number of work disparities, study findings, future directions for research, and use of work disparity terminology. Further, work disparities within the included articles were extracted, and the following information was used to categorize each of them: work disparity comparator groups, work disparity comparator group subdivision, variable of work disparity, and variable of work disparity categorization (see Appendix A for definitions). Other data needed to describe the nature of knowledge in the study of work disparities related to health and well-being were also extracted: study rationale associated with health and wellness, the type of health and/or well-being described within the study rationale (i.e., physical health, emotional health, mental health, and general health), and whether health and well-being were analyzed in relation to work disparities as part of the study analysis and/or using external literature.

### 2.6. Quality Appraisal

A quality appraisal of the qualitative and quantitative articles was conducted using a modified version of Hawker and Paynes’s quality appraisal criteria [30]. The tool consists of nine subscales to rate articles: (1) abstract and title; (2) introduction and aims; (3) method and data; (4) sampling; (5) data analysis; (6) ethics and bias; (7) results; (8) transferability and generalizability; and (9) implications and usefulness. The items in each subscale were rated on a 4-point scale, with ratings of very poor (1), poor (2), fair (3), and good (4). Each article received a total score within a range of 9–36; a score of 28–36 was associated with good quality, 20–27 for fair quality, 10–20 for poor quality, and less than 10 for very poor-quality articles [30]. Ratings were independently assigned by one author and reviewed by at least one other author for consensus (L.S., M.M., and K.N.). Disagreements were to be resolved through discussion, although none occurred. Articles in this review were not excluded based on study quality but were utilized to inform the overarching findings of the review.

### 2.7. Data Synthesis

Results were organized to describe the nature and extent of work disparities found using narrative description and tabular formatting. This included categorizing the data according to (1) study characteristics; (2) work disparity traits; and (3) health and well-being features. Descriptive statistics of individual variables (e.g., frequencies of identified work disparity characteristics) are presented using narrative synthesis.

## 3. Results

### 3.1. Search Results

**Figure 1:**
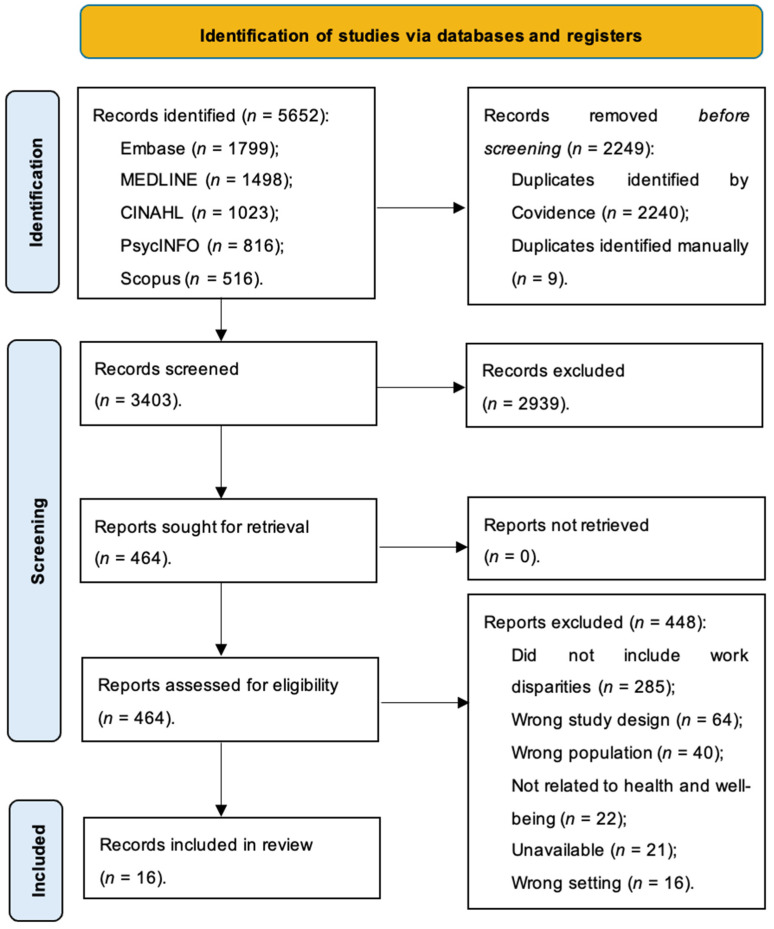
PRISMA Flowchart. (This was derived from [31]).

A total of 5652 records were identified from all databases after both searches. Following de-duplication, title and abstract screening were performed on the remaining records (*n* = 3403) and resulted in 464 articles. Full-text screening was executed on all retrieved studies (*n* = 464). After this process, a total of 16 articles met the inclusion criteria and were included in the present review. See Figure 1 for the PRISMA flowchart diagram.

### 3.2. Eligible Source Characteristics

Sixteen articles published between 1997 and 2024 met the inclusion criteria following screening (see Table 1 for study characteristics). Articles originated from the United States (*n* = 7), the Netherlands (*n* = 2), Sweden (*n* = 2), Germany (*n* = 1), Korea (*n* = 1), Slovenia (*n* = 1), Spain (*n* = 1), and the United Kingdom (*n* = 1). While most sources conducted a cross-sectional survey (*n* = 9), studies also conducted national surveys (*n* = 3), qualitative analyses (*n* = 2), a mixed-methods study (*n* = 1), and a causal model of turnover and a predictive study (*n* = 1). The average number of work disparities within each study was 3.06 (minimum = 1, maximum = 6, SD = 1.62). Most articles (*n* = 13) did not make use of work disparity terminology (e.g., “work disparity”, “inequality”, and “inequity”) to describe the work disparities within that given study. Of the three that did employ such terminology, two articles used “inequalities”, and one used “disparity of working conditions” as descriptors. Hawker and Payne’s (2002) quality appraisal identified that 14 studies were of good quality, two were of fair quality, and none were of poor quality.

### 3.3. Work Disparities

A total of 53 work disparities were identified from the 16 studies that met the inclusion criteria (see Table S3 for the complete list of work disparities). The group identity comparison variable across all identified work disparities was as follows: professional status (*n* = 14), location of work (*n* = 10), age (*n* = 8), race (*n* = 7), gender (*n* = 6), marital status (*n* = 3), experience (*n* = 2), education status (*n* = 1), geographic location (*n* = 1), and number of children (*n* = 1). Out of the 53 work disparities, 19 were classified as a “work opportunity”, 18 as a “workplace treatment”, and 8 as being related to “job security” and “work compensation”, respectively. Comparator group subdivisions of work disparities were classified as 7 “simple intragroup”, 14 “simple intergroup”, 21 “mixed intragroup”, and 11 “mixed intergroup” disparities. A matrix presenting the number of work disparities with each comparator group subdivision in comparison to each variable of the disparity category is provided in Table 2.

### 3.4. Health and Well-Being

The background rationale for each study in this review noted that nurses experienced several types of health conditions and/or disruptions in relation to well-being in LTC, specifically, the types of health and well-being emphasizing physical health (*n* = 8), general health (*n* = 4), emotional health (*n* = 4), and mental health (*n* = 8). Not all studies investigated health and well-being links to work disparity. Four studies investigated the association of an identified work disparity with a variable of health and well-being as part of their analysis. Five studies used the external literature to describe the association between a variable of health and well-being and an identified work disparity. See Table S4 for the health and well-being data for all included studies.

## 4. Discussion

This scoping review describes work disparities experienced by nurses in the LTC sector within the research literature. A total of 16 articles conducted in seven countries over 26 years (1997–2024) met the inclusion criteria (see Table 1 for study characteristics). Further, 53 work disparities were identified, extracted, and categorized from these articles (see Table S3). The results provide insight into the complexity of examining work disparities for nurses in LTC, what is known, how work disparities have been studied, and what is needed to advance research on the work disparities of nurses in the LTC sector.

The nature of the research findings from this review suggests that the concept of work disparities is complex, difficult to understand, and challenging to research. Work disparities have not been consistently expressed using common terminology across the nursing literature in LTC [16]. Within the present review, only three articles used “work disparity” and/or similar terminology (i.e., “inequalities” and “disparity of working conditions”) to describe the work disparities present within their studies, while the remaining articles (*n* = 13) did not. Moreover, ways to categorize and analyze different types of work disparities with the nuance and reflection of different workplace settings and contexts are predominantly absent within the research literature for nurses in LTC. In response, the authors of the present scoping review were required to create a conceptual framework that provided definitions and categorizations (see [16]) to allow for the identification and exploration of work disparities. With work disparities being a complex yet necessary concept to analyze, the absence of a mechanism to conduct research and understand work disparities suggests that a gap is present on this topic and that increased research to develop such frameworks is required to allow for the understanding of the day-to-day experiences and inequities experienced by nurses in LTC.

The use of the work disparity conceptual framework terminology [16] supported the framing of the 53 work disparities identified from the 16 included studies into four meso-level work disparity categories (i.e., work opportunities [*n* = 19], work treatment [*n* = 18], job security [*n* = 8], and work compensation [*n* = 8]). These categories offer an initial and novel starting point to make the knowledge of the work disparities in the LTC sector more readily understood and apparent in the research. These results highlight that work opportunities (i.e., inequities for opportunities for career growth or growth within the workplace) and workplace treatment (i.e., differences in workplace treatment of nurses) are the most frequently studied work disparities within the research literature regarding nurses in LTC. Studies exploring these two categories consequently provide an initial direction for LTC organizations to begin to investigate, address, and focus on opportunities for growth and emphasizing improvements for nurses performing care work in LTC. The other two categories, work compensation (pay and benefits) and job security (work retention and recruitment), were present to a much lesser extent within the literature. While the rationale behind why these categories were disproportionately explored remains unclear, further research that focuses on the nature of these relationships may yield opportunities for greater fairness and access to benefits that can make an impact on the work lives of nurses. In addition, future attention to examining these four categories at the macro level may provide a more consistent approach to investigating work disparities. Shaw and colleagues [46,47,48] and Ndugga et al. [18] have advocated that research into macro-level issues is warranted to advance systemic and contextual knowledge of how systemic inequities manifest work disparities that emerge at the meso level. Specifically, at the macro level, more examination of inequities is needed in relation to the socioeconomic determinants of health (e.g., education, employment status, income, and housing) [21], as well as social factors, such as discrimination [18] and the lack of access to full-time work in LTC. Thus, to advance knowledge of the complexities of work disparities that are experienced by groups of nurses in LTC, an intersectoral approach, along with appropriate mixed-methods and qualitative designs, will be needed to shed light on where there are opportunities to make impactful changes in this sector.

The nature and frequency of the variables used to compare groups within the 53 identified work disparities were categorized as part of this analysis. The most frequently mentioned variables are professional status (*n* = 14), location of work (*n* = 10), age (*n* = 8), race (*n* = 7), and gender (*n* = 6); and the least mentioned was marital status (*n* = 3), experience (*n* = 2), education status (*n* = 1), geographic location (*n* = 1), and number of children (*n* = 1). These categories suggest that a diversity of issues is being investigated within research related to nurses in LTC, all of which were reported to be relevant with regard to the treatment and experiences of nurses in LTC in the 16 studies in this review. In these studies, professional status was used to compare groups. Given that the professional status of nursing staff in long-term care varies and that there can be shifts in education (e.g., through bridging programs from RPNs/LPNs to RNs [49]), the variables of professional status, experience, and educational status will continue to be needed to identify emergent disparities that may arise due to these changes for nurses in LTC. While comparing groups of nurses using variables such as work location, geographical location, or number of children may support a contextual understanding of work and life disparities, the results suggest that they have not been frequently investigated. However, the results from this review also suggest that specific variables that impact nurses in LTC have not been adequately investigated to date, and others may be needed, including, but not limited to, socioeconomic status, sexual orientation, religious identity, and physical ability. Ndugga et al. [18] argued for an increased focus on these variables, with a specific emphasis on social determinants of health, to deepen the understanding of the lived experiences of nurses. Recognizing the importance of diversity, equity, and inclusivity within LTC contexts at policy and community of practice levels [50,51], these findings suggest that research needs to be conducted on a broader scale and account for a larger diversity of backgrounds when investigating work disparities in relation to nurses in LTC [18]. This was also emphasized by Boamah et al. [8], who underscored concerns about the growth of intersectional inequalities in the context of LTC for healthcare workers, including nurses, during and post the COVID-19 pandemic, such as “increased work precarity, social marginalization, workplace inequalities, and intersectional racism” (p. 1130).

Adhering to the conceptual framework proposed by Shaw et al. [16], this scoping review categorized work disparities in accordance with the four ways that nurses in LTC (i.e., target group) may be compared with other groups within the healthcare system. Specifically, work disparities were categorized as simple intragroup (*n* = 7), simple intergroup (*n* = 14), mixed intragroup (*n* = 21), and mixed intergroup disparities (*n* = 11; see Table S3). The results revealed that work disparities of nurses in LTC were studied more often as part of a mixed group with other healthcare workers (i.e., as part of mixed intragroup/intergroup disparities [*n* = 32]) and less often as isolated, homogenous groups solely including nurses from LTC (i.e., as simple intragroup/intergroup disparities [*n* = 21]). While the value of each type of comparison has not been evaluated, such observations suggest that definitive conclusions on the experiences of nurses in LTC are difficult to ascertain because most research on work disparities has included nurses in LTC as part of a larger group. Future research, where experiences solely within nurses in LTC (i.e., simple intragroup disparities) or solely between nurses in LTC and another healthcare group (i.e., simple intergroup disparities) are analyzed, will be beneficial in enriching work disparity literature. The matrix of comparator group subdivisions of work disparities (see explanation in [16]) may support such study designs and help provide clarity on the framing of the comparator groups in studies of work disparities.

Key areas of health concerns related to nurses in LTC informed the need for an examination of differences and inequities. The most salient health concerns were physical health (*n* = 8), general health (*n* = 4), emotional health (*n* = 4), and mental health (*n* = 8; see Table S4). Despite the importance of health and well-being as drivers that underscored the research investigations, only four out of the 16 included studies examined specific health outcomes or used measures as part of their research design (Table S4). Such results suggest that insufficient information exists on how to identify the health impact of work disparities on workers. This finding is consistent with Le’s [22] observations and suggests that more knowledge on how to approach health surveillance, which considers the intersection of the worker, the workplace, and the broader nursing context, is necessary. Research related to the resilience of nurses is required to support the understanding of how resilience links to health in relationship to challenging work demands in the LTC sector [52,53,54]. For instance, the focus on building individual resilience has prompted a breadth of training for nurses in LTC [55,56,57]. However, an emphasis on resilience is only one aspect of supporting better health and the retention of nurses in this sector. More knowledge on the types of work disparities that impact the health and well-being of nursing staff in LTC, beyond individual resilience, may also inform opportunities to improve the retention of groups of nurses working in the LTC sector. For instance, Boamah and colleagues [8] proposed other strategies to promote the health and well-being of nurses and other workers in LTC (e.g., the creation of safe and healthy work environments, provision of appropriate recognition and development of creative and innovative programs). These suggestions align with addressing disparities found in this review related to work treatment (unfair work demands and excessive work strain) and work opportunities (unequal access to professional education or career growth). Finally, there is a consistent call for an intersectoral approach [8,16,18,22] to further address the complexities and challenges of work disparities and the negative health outcomes for nurses in LTC that lead to imbalances in work and home life. Intersectoral perspectives and knowledge are needed to develop and advance healthy workplaces where nurses in LTC can thrive, such as from occupational health and safety organizations, ministries of health and LTC administration, researchers in workforce planning, organizational health and well-being, occupational science, physical therapy science, and nursing science, as well as nursing professional, regulatory, and/or labor associations. All these perspectives can better elucidate the association between health and work disparities, such that a holistic understanding of the experiences of nurses in LTC may be considered and responded to on a higher level.

Key perspectives needed in the change process are the nurses themselves; they have an integral role in informing practice and policies that can help achieve equitable practices and reduce work disparities. Nurses possess the strongest ability to create change and, consequently, need to be engaged in the policy and social transformation on health and well-being in LTC nursing work and life balance [58]. Nurses may advocate for and enable change within all four categories described within the present scoping review (i.e., work opportunities, work compensation, job security, and workplace treatment). For example, to improve ‘work opportunities’, nurses can participate in reviewing and identifying educational topics for meaningful professional development, specifically tailoring such resources to the needs of individuals or groups of nurses. In the area of ‘work compensation’, nurses may advocate for benefits needed to support a work and life balance (e.g., daycare or respite care for family members, access to employee assistance for compassion fatigue, or wellness supports). With regard to ‘work treatment’, participation in the evaluation of work schedules to identify ways to improve flexibility yet also support a balance of heavy and light work demands may reduce exposure to heavier demands and reduce risks related to musculoskeletal strains. When advocating for ‘job security,’ nurses can work with their nursing associations and researchers to support and use evidence to advocate for change in creating more secure work opportunities that are contextually appropriate in various LTC settings. The present scoping review acts as an initial step in enabling such advocacy by presenting the extent and nature of research on work disparities in all four of these categories of the literature. By identifying areas of deficits or gaps, this also informs directions for future investigation and advocacy. For instance, a need for research that uses an intersectoral approach to examine macro and meso factors to contribute to the knowledge of work disparities in LTC, as well as the need for nurses to share their lived experiences to inform deeper insights into the impact of work disparities on nurse health and well-being. The need for advocacy is critical to begin the work of increasing awareness of areas of where change can begin that can respond to the health and work balance needs of nurses in LTC.

*Strengths and Limitations*

The strength of this review study is the use of a novel conceptual framework to structure the findings that were specifically developed to understand work disparities in the LTC for nurses [16]. The terminology supported the search, selection, and data extraction needed to inform a description of what exists in the literature that relates to the health and well-being of nurses and how work disparities can be understood within the workplace, as well as guide future studies on such disparities. Another strength is the examination of the quality of the studies using Hawker and Paynes’s quality appraisal criteria. Rating indicated that studies were either good (*n* = 14) or fair (*n* = 2) quality, with none being of poor quality, thereby suggesting that research has been conducted with rigor when examining the complex phenomenon of work disparities experienced by nurses in LTC. Thus, the results of these studies support a base of knowledge into work disparities experienced by nurses evaluated within LTC and suggest that insights from the present analysis may be built upon by future researchers when designing studies to evaluate the impact of and relationships of work disparities on health outcomes. Another strength of this review is the comprehensive search of multiple databases using a librarian’s support and multiple reviewers, which supported the integrity of the search and selection processes. A strength of the included literature is that this review focused specifically on knowledge of inequities of licensed nurses in the LTC sector, which revealed a realm of meso-level work disparities that may impact the health of this group of nurses.

Alternatively, this review did not include the gray literature, which may have limited other information that could expand knowledge of macro-level factors and how they contribute to meso-factors that shape work disparities in LTC for groups of nurses. Further, studies were limited to articles available in English; therefore, relevant knowledge may have been missed, and future work is needed to expand the search to other languages. This review was limited to identifying potential gaps in the literature, and thus, a critical synthesis of geographical differences in the experience of work disparities was not conducted. However, geographical representation was discovered to be restricted to Korea, Europe, the USA, the UK, and Scandinavia. Given that work disparities experienced by nurses in LTC are situated globally and shortages of nurses are forecasted within other regions (e.g., Canada), more studies of work disparities and their impact on groups of nurses are warranted on a global scale. This international research focus will support the future synthesis of common disparities and examinations of contextual comparisons by settings and regions, enabling organizations to target approaches to fairness that can make a difference in the mental, physical, and emotional health of nurses.

## 5. Conclusions

This scoping review revealed the extent and nature of knowledge present in the research literature with regard to meso-level work disparities experienced by nurses in LTC. The results suggest that work disparities are a complex phenomenon to analyze and reveal gaps in the literature with studies analyzing work disparities experienced by nurses in LTC. Further examination of macro-level factors is indicated to move towards an intersectoral approach in the study of the complex issue of work disparities. Research of nurses’ lived experience in relation to poor health due to work disparities can provide deeper insights into the complex relationship of macro- and meso- level factors that influence health and work–life balance. These new discoveries will support the development of study designs that examine the impact of social and economic inequalities, along with unfair practices, in the workplace on the health of groups of nurses in LTC. Future research is warranted to expand the meso- and macro-conceptual understandings, tools, and instruments with regard to examining work disparities. Furthermore, more studies using the lenses of lived experience or intersectionality will increase the scope of understanding of the links between work disparities, nurse health, and retention in LTC. In doing so, qualitative and mixed methods studies may be helpful for informing changes in workplace health, safety, equity, and employment policy and practices. These changes are indicated to support opportunities for all nurses to participate in professional development, improve balanced work demands, and foster the prioritization of nurse health with the goals of increasing retention and facilitating healthier, safer, and more inclusive work environments for nurses in LTC.

## Figures and Tables

**Table 1:** Included study characteristics.

Author (Year)	Country *	Type of Study	Aim	Study Sample	No. of WDs	Use of WD Terms †	Quality Appraisal Score ‡
Bae & Brewer (2010) [9]	USA	National Survey	To describe (a) the nature and occurrence of mandatory and voluntary overtime for nurses as well as nurses’ paid on-call hours and (b) the associations with mandatory overtime regulations.	*n* = 6158 RNs	6	No	30 (G)
Banaszak-Holl et al. (2015) [32]	USA	Cross-sectional Survey	To examine how organizational culture in nursing homes affects staff turnover.	*n* = 419 NH administrators	1	No	31 (G)
Baughman et al. (2022) [33]	USA	National Survey	To estimate the rate at which direct care workers and nurses hold multiple jobs, the factors associated with multiple job holding, and the mix of employment across settings for those who do hold a second job.	*n* = 38,933 Direct care workers/Nurses	5	No	33 (G)
Blanco-Donoso et al. (2021) [12]	SPA	Cross-sectional Survey	To analyze the psychological consequences of the COVID-19 pandemic on nursing home workers, as well as the influence of certain related stressors and job resources.	*n* = 228 NH workers	2	No	33 (G)
Bratt & Gautun (2018) [34]	UK	Cross-sectional Survey	To investigate the prevalence of nurses’ wishes to leave work in elderly care services and to explain differences between younger and older nurses.	*n* = 4945 Nurses	2	No	35 (G)
Castle et al. (2006) [35]	USA	Cross-sectional Survey	To examine the job satisfaction scores of these caregivers and what characteristics of these caregivers are associated with job satisfaction.	*n* = 574 NH caregivers	6	No	33 (G)
Dill & Duffy (2022) [36]	USA	National Survey	To describe how structural racism and sexism shape the employment trajectories of Black women in the US healthcare system.	*n* = 125,800 Healthcare workers	2	No	32 (G)
Duijis et al. (2023) [37]	NET	Qualitative Analysis	To understand self-employed long-term-care workers’ experiences of precariousness and to unravel how their experiences are shaped at the intersection of gender, class, race, migration, and age.	*n* = 23 Self-employed nurses and NAs in LTC	2	Yes; ‘Inequalities’	36 (G)
Elwér et al. (2012) [38]	SWE	Qualitative Analysis	To analyze what gender (in)equality means for the employees at a woman-dominated workplace and to discuss possible implications for health experiences.	*n* = 45 NH workers	4	Yes; ‘Inequalities’	34 (G)
Hasson & Arnetz (2008) [39]	SWE	Cross-sectional Survey	To compare the perceptions of competence, work strain, and work satisfaction among nursing staff providing care to older people in nursing homes and home-based care; to examine determinants of work satisfaction in both care settings.	*n* = 863 Nursing staff	2	No	32 (G)
Kiyak et al. (1997) [40]	USA	Modeling predictive study	To integrate previous approaches to studying turnover in organizations serving elderly persons.	*n* = 308 NH/community agency employees	3	No	26 (F)
Krsnik & Erjavec (2023) [41]	SLO	Cross-sectional Survey	To use multivariate analysis to identify the macro-, meso-, and micro-level factors that influence LTC workers’ turnover in Slovenia, a typical Central and Eastern European country.	*n* = 452 LTC workers	3	No	25 (F)
Min et al. (2022) [42]	KOR	Mixed-Methods	To identify the factors associated with retention intention among registered nurses in South Korean nursing homes.	*n* = 155 RNs	4	No	34 (G)
Rahnfeld et al. (2016) [43]	GER	Cross-sectional Survey	To examine mediators in the relationship between care setting and turnover intentions.	*n* = 278 RNs and NAs	1	No	29 (G)
TenHoeve et al. (2024) [44]	NET	Cross-sectional Survey	To explore motivation, organizational climate, work engagement, and related factors within the practice environment of nurse practitioners.	*n* = 586 NPs	4	No	33 (G)
Zhang et al. (2016) [45]	USA	Cross-sectional Survey	To evaluate the association between working conditions and mental health among different nursing groups and examine the potential moderating effect of job groups on this association.	*n* = 1129 Nursing staff	3	Yes; ‘Disparity of working conditions’	32 (G)

Note. WD: Work Disparities; RN: Registered Nurses; NP: Nurse Practitioner; NA: Nursing Aide; LTC: Long-Term Care; NH: Nursing Home. * GER: Germany; KOR: Korea; NET: Netherlands; SLO: Slovenia; SPA: Spain; SWE: Sweden; UK: United Kingdom; USA: United States. † If yes, the article used work disparity terminology (e.g., “work disparity”, “inequality”, and “inequity”) to describe the work disparities within that given study. ‡ G: Good Quality (28–36 points); F: Fair Quality (20–27 points); P: Poor Quality (10–20 points).

**Table 2:** Matrix presenting categories of work disparities (*n* = 53) from the literature based on nurses in LTC.

		Comparator Group Subdivision				
		S-INTRA	S-INTER	M-INTRA	M-INTER	Total
Variable of Work Disparity Categorization	Job Security	0	2	3	3	8
	Work Compensation	1	2	3	2	8
	Work Opportunities	6	4	6	3	19
	Workplace Treatment	0	6	9	3	18
	Total	7	14	21	11	53

*Note*. S-INTRA: Simple Intragroup Disparity; S-INTER: Simple Intergroup Disparity; M-INTRA: Mixed Intragroup Disparity; M-INTER: Mixed Intergroup Disparity.

## Data Availability

The original contributions presented in the study are included in the article/Supplementary Materials; further inquiries can be directed to the corresponding author.

## Supplementary Materials

The following supporting information can be downloaded at https://www.mdpi.com/article/10.3390/healthcare12202065/s1. Table S1. Preferred Reporting Items for Systematic Reviews and Meta-Analyses Extension for Scoping Reviews (PRISMA-ScR) Checklist; Table S2. MEDLINE (Ovid) Search Strategy for Work Disparities Experienced by Nurses in Long-Term Care (LTC) conducted on 25 January 2024; Table S3. Work Disparities Identified from Included Studies; Table S4. Health and Well-Being Association within Included Articles.

## Appendix A

**Table A1:** Work disparity conceptual framework terminology.

Term	Definition
1. Variable of work disparity	Any defined factor, construct, or characteristic of inequality or factor of unfairness/difference. Four categories were identified based on a sample of the literature on work disparities experienced in LTC for nurses: job security, work compensation, work opportunities, and/or workplace treatment.
1a. Measure of disparity	Values that quantitatively/qualitatively measure, examine, and/or explore variables of work disparity.
1b. Categories	Examples
1b(i). Job Security	Hiring /dismissal practices, Intent to leave, Retention, Recruitment
1b(ii). Work Compensation	Wages, Benefits, Paid-vacation days, Health insurance
1b(iii). Work Opportunities	Ability to be promoted in an organization, Development opportunities, Exposure to challenging/meaningful work, Amount of work provided
1b(iv). Workplace Treatment	Discrimination, Expectations/demands of performance (e.g., emotional labor, cognitive labor demands), Recognition of contributions, Work satisfaction
2. Comparator Group	A group of individuals (workers) composed of members of the workforce.
2a. Group identity	The identity associated with each individual comparator group (e.g., gender, race, socioeconomic status, residency status, educational status, profession, indigenous status, work setting, age, sexuality, disability, height, identities 2SLGBTQAI plus, weight, ethnicity, and religion).
2b. Group identity Comparison Variable	The group identity that differentiates one comparator group from the other for a specific work disparity (e.g., if a work disparity is identified between old and young workers, the group identity comparison variable would be ‘age’). The group identity comparison variable must be related to the demographic variables and must not be subjective in nature (e.g., job satisfaction cannot be a group identity comparison variable because it is a subjective measure).

Note. Reprinted with permission from IOS Press.
