# Supplementary material for: Work Disparities and the Health of Nurses in Long-Term Care: A Scoping Review
Source: Healthcare (Basel). 2024 Oct 17;12(20):2065. doi: 10.3390/healthcare12202065 (PMC11508128; doi:10.3390/healthcare12202065)
Supplement: Supplementary file 1 [file healthcare-12-02065-s001.zip › healthcare-3214373-supplementary.pdf]

Supplementary Files

Table S1. Preferred Reporting Items for Systematic Reviews and Meta-Analyses Extension for Scoping Reviews (PRISMA-ScR) Checklist

SECTION	ITEM	PRISMA-ScR CHECKLIST ITEM	REPORTED ON PAGE #
**TITLE**			
Title	1	Identify the report as a scoping review.	1
**ABSTRACT**			
Structured summary	2	Provide a structured summary that includes (as applicable): background, objectives, eligibility criteria, sources of evidence, charting methods, results, and conclusions that relate to the review questions and objectives.	1
**INTRODUCTION**			
Rationale	3	Describe the rationale for the review in the context of what is already known. Explain why the review questions/objectives lend themselves to a scoping review approach.	1-2
Objectives	4	Provide an explicit statement of the questions and objectives being addressed with reference to their key elements (e.g., population or participants, concepts, and context) or other relevant key elements used to conceptualize the review questions and/or objectives.	2
**METHODS**			
Protocol and registration	5	Indicate whether a review protocol exists; state if and where it can be accessed (e.g., a Web address); and if available, provide registration information, including the registration number.	3
Eligibility criteria	6	Specify characteristics of the sources of evidence used as eligibility criteria (e.g., years considered, language, and publication status), and provide a rationale.	3-4
Information sources*	7	Describe all information sources in the search (e.g., databases with dates of coverage and contact with authors to identify additional sources), as well as the date the most recent search was executed.	3
Search	8	Present the full electronic search strategy for at least 1 database, including any limits used, such that it could be repeated.	3, Table S2

SECTION	ITEM	PRISMA-ScR CHECKLIST ITEM	REPORTED ON PAGE #
Selection of sources of evidence†	9	State the process for selecting sources of evidence (i.e., screening and eligibility) included in the scoping review.	3-4
Data charting process‡	10	Describe the methods of charting data from the included sources of evidence (e.g., calibrated forms or forms that have been tested by the team before their use, and whether data charting was done independently or in duplicate) and any processes for obtaining and confirming data from investigators.	4
Data items	11	List and define all variables for which data were sought and any assumptions and simplifications made.	4
Critical appraisal of individual sources of evidence§	12	If done, provide a rationale for conducting a critical appraisal of included sources of evidence; describe the methods used and how this information was used in any data synthesis (if appropriate).	4-5
Synthesis of results	13	Describe the methods of handling and summarizing the data that were charted.	5
**RESULTS**			
Selection of sources of evidence	14	Give numbers of sources of evidence screened, assessed for eligibility, and included in the review, with reasons for exclusions at each stage, ideally using a flow diagram.	5, Figure 1
Characteristics of sources of evidence	15	For each source of evidence, present characteristics for which data were charted and provide the citations.	7, Table 1
Critical appraisal within sources of evidence	16	If done, present data on critical appraisal of included sources of evidence (see item 12).	7, Table 1
Results of individual sources of evidence	17	For each included source of evidence, present the relevant data that were charted that relate to the review questions and objectives.	11, Table S3, Table S4
Synthesis of results	18	Summarize and/or present the charting results as they relate to the review questions and objectives.	11 Table S3, Table S4
**DISCUSSION**			
Summary of evidence	19	Summarize the main results (including an overview of concepts, themes, and types of evidence available), link to the review	11-13

SECTION	ITEM	PRISMA-ScR CHECKLIST ITEM	REPORTED ON PAGE #
		questions and objectives, and consider the relevance to key groups.	
Limitations	20	Discuss the limitations of the scoping review process.	13-14
Conclusions	21	Provide a general interpretation of the results with respect to the review questions and objectives, as well as potential implications and/or next steps.	14
**FUNDING**			
Funding	22	Describe sources of funding for the included sources of evidence, as well as sources of funding for the scoping review. Describe the role of the funders of the scoping review.	14

From: Tricco AC, Lillie E, Zarin W, O'Brien KK, Colquhoun H, Levac D, et al. PRISMA Extension for Scoping Reviews (PRISMAScR): Checklist and Explanation. Ann Intern Med. 2018;169:467–473. doi: 10.7326/M18-0850.

Table S2. MEDLINE (Ovid) Search Strategy for Work Disparities Experienced by Nurses in Long-Term Care (LTC) conducted on January 25, 2024

1: (work* adj3 disparit*).tw.<br>2: (labo*r adj3 disparit*).tw.<br>3: (earning* adj3 disparit*).tw.<br>4: (job adj3 disparit*).tw.<br>5: (employment adj3 disparit*).tw.<br>6: (occupational adj3 inequalit*).tw.<br>7: (employment adj3 inequalit*).tw.<br>8: (work* adj3 inequalit*).tw.<br>9: Job Satisfaction/ or "job satisfaction".tw. or "job dissatisfaction".tw.<br>10: Working Conditions/ or Occupational Stress/<br>11: (wage adj3 disparit*).tw.<br>12: (nurs* adj5 disparit*).tw.<br>13: exploit*.tw.<br>14: Racism/<br>15: (race or racism or racial).tw.<br>16: Poverty/<br>17: poverty.tw.<br>18: (divers* or ethnicity or equity).tw.<br>19: Occupational Health/<br>20:((occupation* or worker*) adj3 (health or wellbeing or safety)).tw.<br>21: 1 or 2 or 3 or 4 or 5 or 6 or 7 or 8 or 9 or 10 or 11 or 12 or 13 or 14 or 15 or 16 or 17 or 18 or 19 or 20<br>22: Long-Term Care/<br>23: Nursing Homes/<br>24: ("care facilit*" or "assist* living" or "nursing home*" or "long term care" or "skilled nursing facilit*" or "home* for the aged" or "aged care facilit*" or "community-based setting*" or "community setting*").tw.<br>25: 22 or 23 or 24<br>26: licensed practical nurses/ or nursing assistants/ or nurses/ or nurse practitioners/ or nursing staff/<br>27: nurs*.tw.<br>28: 26 or 27<br>29: 21 and 25 and 28<br>30: (patient* or resident*).tw.<br>31: 29 not 30

Table S3. Work Disparities Identified from Included Studies

No.	Work Disparity	Group Identity Comparison Variable	Variable of Work Disparity (Categorization*)	Work Disparity Comparator Group Subdivision†
1	"Results show that workers who have the intention to leave in the next six months are, in general, younger. The highest share (32.2%) of workers who intend to leave were aged between 31 and 40, followed by workers aged between 21 and 30 (29.7%) and those aged between 41 and 50 (28.4%)." (p. 7) (Krsnik & Erjavec, 2023) [1]	Age	Intent to Leave (1)	M-INTER
2	"The wish to leave was more prevalent among younger nurses [compared to older nurses]." (p. 1077) (Bratt & Gautun, 2018) [2]	Age	Intent to Leave (1)	M-INTRA
3	"Older workers had expressed a greater intention to stay, and their expectations were consistent with outcomes. Most were, in fact, still on the job 1 year later ($F = 27.86$, $p < 0.0001$)." (p. 235) (Kiyak et al., 1997) [3]	Age	Intent to Leave (1)	M-INTRA
4	"Older workers were slightly more satisfied with pay ($p < .001$)" (p. 8) (Castle et al., 2006) [4]	Age	Satisfaction with Pay (2)	M-INTRA
5	"The older (60+), often white, participants in our study did not perceive self-employment as precarious work. In relation to the first theme, these women were mainly pulled to self-employment to enhance their work pleasure or as a health strategy and could afford to do so." (p. 1808) (Duijis et al., 2023) [5]	Age	Precariousness (4)	M-INTRA
6	"There was also an age difference, as the older women took more responsibility and to some extent also took the responsibility to tell their co-workers what to do." (p. 6) (Elwér et al., 2012) [6]	Age	Work Responsibility (4)	M-INTRA
7	"Satisfaction was highest among workers who were older" (p. 237) (Kiyak et al., 1997) [3]	Age	Job Satisfaction (4)	M-INTRA

8	"[P]articipants aged 45 or over scored significantly higher on autonomy satisfaction (p= .006); autonomy frustration (p= .049); and competence satisfaction (p= .017)." (p. 548) (TenHoeve et al., 2024) [7]	Age	Autonomy Satisfaction, Autonomy Frustration, Competence Satisfaction (4)	M-INTRA
9	"Retention intention was higher in the group with a BSN or above (β = 3.63, p = 0.014)" (p. 464) (Min et al., 2022) [8]	Education Status	Retention Intension (1)	M-INTRA
10	"Similarly, workers who had been working in the LTC sector for a shorter period of time would rather leave their work than those who had worked in this sector for more than 20 years." (p. 7) (Krsnik & Erjavec, 2023) [1]	Experience	Intent to Leave (1)	M-INTER
11	"Results of the nonparametric tests revealed that respondents who were working 6 years or longer as an NP scored significantly higher on all subscales of the BPNSFS (motivation in the workplace)." (p. 548-549) (TenHoeve et al., 2024) [7]	Experience	Motivation in the Workplace (4)	M-INTRA
12	"Male nurses worked more mandatory overtime and voluntary overtime than female nurses." (p. 105) (Bae & Brewer, 2011) [9]	Gender	Mandatory and Voluntary Overtime (3)	M-INTRA
13	"The participants said that women at the workplace were specialized in more work tasks than the men, which was described as adding to their workload and contributing to increased risk of vulnerability, tiredness and stress." (p. 5) (Elwér et al., 2012) [6]	Gender	Workload (3)	M-INTRA
14	"Female[s] […] are less likely to hold second jobs." (p. 155) (Baughman et al., 2022) [10]	Gender	Second Job (3)	S-INTRA
15	"We found that males were less satisfied with work than females" (p. 8) (Castle et al., 2006) [4]	Gender	Job Satisfaction (4)	M-INTRA
16	"Some of the participants felt that less was expected of their male colleagues and that the men in the workgroup got more appreciation, which was considered unfair." (p. 5) (Elwér et al., 2012) [6]	Gender	Work Expectations (4)	M-INTRA

17	"Women were often described as taking on more responsibility by seeing what needed to be done and doing it, but also as doing overambitious work at an unwarrantedly high pace." (p. 6) (Elwér et al., 2012) [6]	Gender	Work Responsibility (4)	M-INTRA
18	"Between rural and urban areas, nurses working in urban areas reported higher levels of voluntary overtime." (p. 105) (Bae & Brewer, 2012) [9]	Geographic location	Voluntary Overtime (3)	M-INTRA
19	"Based on the organizational form of the institution, workers who had the intention to leave were mainly employed in the public sector (73.0%), especially in a nursing home (63.5%), followed by specialized social welfare institutions (14.9%) and centers for social work (12.2%)." (p. 7) (Krsnik & Erjavec, 2023) [1]	Location of Work	Intent to Leave (1)	M-INTER
20	"The analysis also showed a difference between nursing homes and home nursing, with more nurses wanting to leave in nursing homes (26.5% yes and 23.9% uncertain) than in home nursing (21.6% yes and 26.1% uncertain). The difference between nursing homes and home nursing in the prevalence of wanting to leave was statistically significant (p = .001)." (p. 1077-1078) (Bratt & Gautun, 2018) [2]	Location of Work	Intent to Leave (1)	S-INTER
21	"In terms of voluntary overtime, we found that RNs working in nonfederal nonpsychiatric hospitals reported working a higher percentage of paid on-call hours (6.3%) than nurses working in federal government hospitals (3.5%) and nursing homes (1.8%)." (p. 105) (Bae & Brewer, 2010) [9]	Location of Work	Voluntary Overtime (Paid On-Call Hours) (3)	M-INTER
22	"Nursing home staff on average worked 4 hours longer [compared to community workers]." (p. 229) (Kiyak et al., 1997) [3]	Location of Work	Hours of Work (3)	M-INTER
23	"Among nurses, those in nursing homes are more likely to have second jobs than those in hospitals." (p. 153) (Baughman et al., 2022) [10]	Location of Work	Second Job (3)	S-INTER

24	"Staff in nursing homes in both municipalities rated several aspects of their work significantly more physically strenuous than staff in home care." (p. 476) (Hasson & Arnetz, 2008) [11]	Location of Work	Physical Strain of Work Tasks (4)	M-INTER
25	"Nurses who worked in nursing homes reported working higher percentages of mandatory overtime than those working in hospitals." (p. 106) (Bae & Brewer, 2010) [9]	Location of Work	Mandatory Overtime (4)	S-INTER
26	"Unlike RNs working in hospitals, RNs experienced identity confusion as nursing home experts owing to unclear duties. The differences in duties between RNs and nursing assistants were not clearly defined, and most tasks that nurses performed were also performed by nursing assistants." (p. 465) (Min et al., 2022) [8]	Location of Work	Identity Confusion (4)	S-INTER
27	"In line with our predictions, geriatric nurses in nursing homes reported significantly more time pressure (H1a) and more work-related social conflicts (H1b) compared to nurses working in home care." (p. 164-165) (Rahnfeld et al., 2016) [12]	Location of Work	Time Pressure, Work-Related Social Conflicts (4)	S-INTER
28	"NPs working in nursing homes were the least frustrated (M 5.17, SD.91), and NPs working in psychiatry were most frustrated (M 4.60, SD 1.31) with their level of autonomy." (p. 549) (TenHoeve et al., 2024) [7]	Location of Work	Frustration with Level of Autonomy (4)	S-INTER
29	"[M]arried caregivers were less satisfied with […] pay (p = .020)" (p. 8) (Castle et al., 2006)	Marital Status	Satisfaction with Pay (2)	M-INTRA
30	"[M]arried nurses are less likely to hold second jobs." (p. 155) (Baughman et al., 2022) [10]	Marital Status	Second Job (3)	S-INTRA
31	"[M]arried caregivers were less satisfied with […] work (p = .040)" (p. 8) (Castle et al., 2006) [4]	Marital Status	Job Satisfaction (4)	M-INTRA
32	"Those with children […] are more likely [to hold second jobs]." (p. 155) (Baughman et al., 2022) [10]	Number of Children	Second Job (3)	S-INTRA

33	"Turnover rates are lower than found in past but remain significantly higher among Nas [nursing assistants] than among RNs [Registered Nurses] or LPNs [Licensed Practice Nurse]." (p. 462) (Banaszak-Holl et al., 2015) [13]	Professional Status	Turnover (1)	S-INTER
34	"The difference in pay between NPs, the Physician Assistants (PAs) and residents in training appeared to be a source of annoyance: 'In general, I think the gap between an NP's salary and what a doctor earns is disproportionate. And although it is evident that a doctor has a higher level of education, much more consideration should be given to the responsibilities a person has. In other words, if tasks and responsibilities or almost identical, then there should also be equal pay.'" (p. 552) (TenHoeve et al., 2024) [7]	Professional Status	Salary (2)	M-INTER
35	"Nurses have significantly higher wages and earnings than direct care workers." (p. 154) (Baughman et al., 2022) [10]	Professional Status	Wages/Earnings (2)	S-INTER
36	"The salaries of RNs were lower than of those in other health occupations." (p. 465) (Min et al., 2022) [8]	Professional Status	Salary (2)	S-INTRA
37	"NAs were less satisfied with pay than nurses (p = .090)." (p. 8) (Castle et al., 2006) [4]	Professional Status	Satisfaction with Pay (2)	S-INTER
38	"Tukey's post hoc test revealed that these differences were at a significant level between the group of doctors and nurses (Group 1) and the group of other professionals (Group 3), the former experiencing higher levels of workload (M = 3.28) than the latter (M = 3.03)." (p. 249) (Blanco-Donoso et al., 2021) [14]	Professional Status	Workload (3)	M-INTER
39	"RNs worked more day shifts and fewer rotating shifts than NAs and LPNs." (p. 5) (Zhang et al., 2016) [15]	Professional Status	Shifts (3)	M-INTRA
40	"In general, a significantly greater percentage of nurses' aides rated their knowledge as insufficient regarding several of the subject areas compared with practical nurses in both	Professional Status	Self-Rated Knowledge (3)	S-INTER

	care settings in both municipalities." (p. 475) (Hasson & Arnetz, 2008) [11]			
41	"Tukey test suggested that RNs and LPNs reported working longer hours per two weeks than did NAs. LPNs reported longer work shifts than did NAs" (p. 5) (Zhang et al., 2016) [15]	Professional Status	Hours of Work (3)	S-INTER
42	"Nurses […] work almost 30% more hours [than direct care workers]." (p. 154) (Baughman et al., 2022) [10]	Professional Status	Amount Worked (3)	S-INTER
43	"There was no scope for career development [for RNs compared to other health occupations]." (p. 465) (Min et al., 2022) [8]	Professional Status	Career Development (3)	S-INTRA
44	"It was also observed that the group of nurse aides perceived more support from the supervisor (M = 2.93) than the group of doctors and nurses (M = 2.58)." (p. 249) (Blanco-Donoso et al., 2021) [14]	Professional Status	Support from Supervisor (4)	M-INTER
45	"NAs [...] reported higher physical demands, lower decision latitude, and lower social support than LPNs and RNs" (p. 5) (Zhang et al., 2016) [15]	Professional Status	Physical Demands, Decision Latitude, Social Support (4)	S-INTER
46	"NAs […] were relatively more satisfied with the work ($p < .001$) [compared to nurses]." (p. 8) (Castle et al., 2006) [4]	Professional Status	Job Satisfaction (4)	S-INTER
47	"Black women are more overrepresented in health care and more concentrated in the lowest-wage direct care jobs (licensed practical nurse and aide occupations) than any other racial or ethnic group of women (and all men)." (p. 269) (Dill & Duff, 2022) [16]	Race	Representation in Lowest-Paying Jobs (2)	M-INTER
48	"White nurses worked less […] paid on-call hours than non-White nurses." (p. 105) (Bae & Brewer, 2013) [9]	Race	Paid On-Call Hours (2)	M-INTRA
49	"Lower-educated care workers of colour mentioned how they lacked access to career opportunities as a hired employee. One participant, for example, shared how her white colleagues obstructed her education, which	Race	Career Opportunities (3)	M-INTRA

	caused her to leave hired employment straight after graduation." (p. 1808) (Duijis et al., 2023) [5]			
50	"Black nurses are more likely [to hold second jobs]." (p. 155) (Baughman et al., 2022) [10]	Race	Second Job (3)	S-INTRA
51	"Black nurses are more likely to hold second jobs than White nurses." (p. 154) (Baughman et al., 2022) [10]	Race	Second Job (3)	S-INTRA
52	"White nurses worked less mandatory overtime, voluntary overtime, […] than non-White nurses." (p. 105) (Bae & Brewer, 2010) [9]	Race	Mandatory Overtime and Voluntary Overtime (3)	M-INTRA
53	"Black women are more likely than any other group to be employed in long-term care and are the only group for which the predicted probability of working in long-term care is higher than in other settings." (p. 268) (Dill & Duff, 2022) [16]	Race	Representation in Long-Term Care (4)	M-INTER

* 1: Job Security; 2: Work Compensation; 3: Work Opportunities; 4: Workplace Treatment

† S-INTRA: Simple Intragroup Disparity; S-INTER: Simple Intergroup Disparity; M-INTRA: Mixed Intragroup Disparity; M-INTER: Mixed Intergroup Disparity

Table S4. Health and Well-Being Association within Included Articles

Article	Study Rationale		WD/H&W Association	
	Quote	H&W Type*	Explored as part of analysis †	External literature ‡
Bae & Brewer (2010) [9]	"Increased overtime may also increase nurse injuries. Trinkoff, Le, GeigerBrown, Lipscomb, and Lang (2006) found that excessive use of overtime increased nurse musculoskeletal problems. Excessive use of overtime has been found to lead to nurse needlestick injuries (Clarke, Rockett, Sloane, & Aiken, 2002). Nurses working more hours were found on average to be more fatigued, have more health complaints, and be less satisfied." (p. 99)	PHYS	No	No
Banaszak-Holl et al. (2015) [13]	"Researchers have argued that the nursing home industry experiences high rates of turnover because working conditions can be both emotionally and physically challenging." (p. 463)	EMO, PHYS	No	No
Baughman et al. (2022) [10]	"This [multiple jobs] regularly puts them in extremely close proximity to patients, and reports suggest that nursing home staff have had even less access to personal protective equipment (PPE) than hospital staff (Rau, 2020; Whoriskey et al., 2020). Second, U.S. nursing homes themselves are generally poorly set up to contain the spread of contagious disease." (p. 152)	PHYS	No	No
Blanco-Donoso et al. (2021) [14]	"The high number of infections and deaths within nursing homes, witnessing the social isolation of the residents and its emotional consequences, has been a severe blow to nursing home employees (Armitage & Nellums, 2020; Fischer et al., 2020). These workers have also been subjected to significant social pressure, both from relatives of the residents and from the health authorities in charge (i.e., regional and community health departments). […] The workload and emotional demands in this sector are already particularly high." (p. 245)	EMO, PHYS, MENT	No	Yes

Bratt & Gautun (2018) [2]	"Nurses can have very demanding and strenuous working conditions (e.g. Jourdain & Chênevert, 2010; Van der Heijden et al., 2010) and it seems likely that emotional and physical stress due to heavy workload and time pressure, along with demands made by superiors, possibly made worse by a non-supportive working environment, can lead to burnout and nurses' decisions to quit their jobs." (p. 1075)	EMO, PHYS, MENT	No	No
Castle et al. (2006) [4]	"Dissatisfied employees often exhibit an unreliable work ethic, including taking unscheduled days off and tardiness. Moreover, dissatisfied employees may also show more aggression towards other workers [5] or residents [3]. In another study of NAs [nursing assistants], job satisfaction was associated with job security, potential for job growth, socialization, and challenging work [20]."	MENT	No	No
Dill & Duffy (2022) [16]	"Overall, health care workers have the highest rates of workplace related injuries of any industry in the United States.[24] Within the workforce, nurse aides and nurses are much more likely to experience workplace-related injuries and stress compared with other health care workers.[25] In addition to being exposed to biological agents such as viruses, direct care workers are exposed to heavy lifting of equipment and patients, physical and verbal assault, and a range of high-stress conditions including long hours and night shift work.[26] Black women are more likely to work in those nursing homes and other long-term care settings that are most understaffed and underresourced, leading to greater risk and exposure to injury or infection." (p. 270)	PHYS, MENT	No	No
Duijis et al. (2023) [5]	"In Western-European countries with an ageing society, tensions arise between the financial sustainability of LTC, the health of paid care workers, and societal support for the policy measures (WRR, 2021). The health of healthcare workers is under pressure, but not for all care workers alike. To understand inequities among LTC workers, we need to unravel the intersecting social determinants of health that shape the health and well-being of care workers in LTC." (p. 1800)	GEN	No	No
Elwér et al. (2012) [6]	"Gendered practices of working life create gender inequalities through horizontal and vertical gender segregation in work, which may lead to inequalities in health between women and men. Gender equality could therefore be a key element of health equity in working life." (p. 1)	GEN	Yes	Yes

Hasson & Arnetz (2008) [11]	"High turnover among older people care nursing staff is a major concern in many countries, and has been associated with the lack of competence development, high levels of work strain and stress and low levels of work satisfaction (Hayes et al. 2005). […] Several studies have reported that heavier work loads have led to increased time pressure among nursing staff, resulting in higher stress levels (Jansen et al. 1996, Laamanen et al. 1999, Brulin et al. 2000, Denton et al. 2002, Morgan et al. 2002)." (p. 469)	EMO	No	No
Kiyak et al. (1997) [3]	"The psychological costs to patients and other staff caused by turnover and absenteeism in health care facilities are difficult to assess, but they may be more important than the financial losses incurred in rehiring and retraining (which are significant, as high as four times the departing member's salary, according to Waxman, Carner, and Berkenstock 1984). Both turnover and absenteeism result in increased workload and resentment among the remaining staff who must assume added responsibilities (Breedlove 1993; Rublee 1986)." (p. 224)	MENT	No	No
Krsnik & Erjavec (2023) [1]	"Additionally, due to under-staffing and overburdening of the remaining LTC workers, health and safety risks for employees increase and, in the worst-case scenario, result in accidents with serious human costs [16]. […] However, there are other important factors that could have an impact on staff turnover, such as work-life balance, which is a common challenge for LTC workers because they have demanding work and often work in shifts, meaning they need to balance their professional duties with their private and family lives [18]." (p. 1-2)	GEN	Yes	Yes
Min et al. (2022) [8]	"RNs working in nursing homes are important because they are responsible for older adults' health, safety, and quality of life. […] The decrease in the number of RNs in nursing homes poses a threat to older residents' safety and the quality of care they receive." (p. 460)	PHYS	No	No
Rahnfeld et al. (2016) [12]	"Nurses in home care have more privileged working conditions than those in nursing homes (Nu¨bling et al. 2010; Wendsche et al. 2014). For example, nurses in home care experience more autonomy as well as less physical and psychological demands (e.g. time pressure) (Hasson and Arnetz 2007; Kromark et al. 2009; Tummers et al. 2013)." (p. 160)	PHYS, MENT	Yes	Yes

TenHoeve et al. (2024) [7]	"Work engagement seems to have benefits for both individuals and for organisations. For instance, it is associated with better health and more life satisfaction (Hakanen & Schaufeli, 2012), and with low risk of sickness absences from work (Schaufeli et al., 2009)." (p. 545)	GEN, MENT	No	No
Zhang et al. (2016) [15]	"In addition, nursing staff in nursing homes often experience physical ailments such as musculoskeletal disorders (Miranda, Punnett, Gore, & Boyer, 2011) and mental health problems such as depression (Eriksen, Tambs, & Knardahl, 2006; Muntaner et al., 2006). Mental health of nursing staff in nursing homes merits close attention from employers and researchers because it may influence resident safety and the quality of care provided to residents." (p. 2)	MENT	Yes	Yes

Note. WD; Work Disparities; H&W: Health and Well-Being

**PHYS: Physical Health; EMO: Emotional Health; MENT; Mental Health; GEN: General Health*

† If yes, the article's analysis explored the association between a work disparity, which was identified through the study's investigation, and a health and well-being variable.

‡ If yes, the article used external literature described the association of health and well-being with a work disparity that was identified through the study's analysis.

References

1. Krsnik, S.; Erjavec, K. Influence of Sociodemographic, Organizational, and Social Factors on Turnover Consideration Among Eldercare Workers: A Quantitative Survey. *Int. J. Environ. Res. Public. Health* **2023**, *20*, 6612, doi:10.3390/ijerph20166612.
2. Bratt, C.; Gautun, H. Should I Stay or Should I Go? Nurses' Wishes to Leave Nursing Homes and Home Nursing. *J. Nurs. Manag.* **2018**, *26*, 1074–1082, doi:10.1111/jonm.12639.
3. Kiyak, H.A.; Namazi, K.H.; Kahana, E.F. Job Commitment and Turnover among Women Working in Facilities Serving Older Persons. *Res. Aging* **1997**, *19*, 223–246, doi:10.1177/0164027597192004.
4. Castle, N.G.; Degenholtz, H.; Rosen, J. Determinants of Staff Job Satisfaction of Caregivers in Two Nursing Homes in Pennsylvania. *BMC Health Serv. Res.* **2006**, *6*, 60, doi:10.1186/1472-6963-6-60.
5. Duijs, S.E.; Abma, T.; Plak, O.; Jhingoeri, U.; Abena-Jaspers, Y.; Senoussi, N.; Mazurel, C.; Bourik, Z.; Verdonk, P. Squeezed out: Experienced Precariousness of Self-Employed Care Workers in Residential Long-Term Care, from an Intersectional Perspective. *J. Adv. Nurs.* **2023**, *79*, 1799–1814, doi:10.1111/jan.15470.
6. Elwér, S.; Aléx, L.; Hammarström, A. Gender (in)Equality among Employees in Elder Care: Implications for Health. *Int. J. Equity Health* **2012**, *11*, 1, doi:10.1186/1475-9276-11-1.
7. Ten Hoeve, Y.; Drent, G.; Kastermans, M. Factors Related to Motivation, Organisational Climate and Work Engagement within the Practice Environment of Nurse Practitioners in the Netherlands. *J. Clin. Nurs.* **2024**, *33*, 543–558, doi:10.1111/jocn.16914.
8. Min, D.; Cho, E.; Kim, G.S.; Lee, K.H.; Yoon, J.Y.; Kim, H.J.; Choi, M.H. Factors Associated with Retention Intention of Registered Nurses in Korean Nursing Homes. *Int. Nurs. Rev.* **2022**, *69*, 459–469, doi:10.1111/inr.12754.
9. Bae, S.-H.; Brewer, C. Mandatory Overtime Regulations and Nurse Overtime. *Policy Polit. Nurs. Pract.* **2010**, *11*, 99–107, doi:10.1177/1527154410382300.
10. Baughman, R.A.; Stanley, B.; Smith, K.E. Second Job Holding Among Direct Care Workers and Nurses: Implications for COVID-19 Transmission in Long-Term Care. *Med. Care Res. Rev. MCRR* **2022**, *79*, 151–160, doi:10.1177/1077558720974129.
11. Hasson, H.; Arnetz, J.E. Nursing Staff Competence, Work Strain, Stress and Satisfaction in Elderly Care: A Comparison of Home-Based Care and Nursing Homes. *J. Clin. Nurs.* **2008**, *17*, 468–481, doi:10.1111/j.1365-2702.2006.01803.x.
12. Rahnfeld, M.; Wendsche, J.; Ihle, A.; Müller, S.R.; Kliegel, M. Uncovering the Care Setting-Turnover Intention Relationship of Geriatric Nurses. *Eur. J. Ageing* **2016**, *13*, 159–169, doi:10.1007/s10433-016-0362-7.
13. Banaszak-Holl, J.; Castle, N.G.; Lin, M.K.; Shrivastwa, N.; Spreitzer, G. The Role of Organizational Culture in Retaining Nursing Workforce. *The Gerontologist* **2015**, *55*, 462–471, doi:10.1093/geront/gnt129.
14. Blanco-Donoso, L.M.; Moreno-Jiménez, J.; Amutio, A.; Gallego-Alberto, L.; Moreno-Jiménez, B.; Garrosa, E. Stressors, Job Resources, Fear of Contagion, and Secondary Traumatic Stress Among Nursing Home Workers in Face of the COVID-19: The Case of Spain. *J. Appl. Gerontol.* **2021**, *40*, 244–256, doi:10.1177/0733464820964153.

15. Zhang, Y.; Punnett, L.; Mawn, B.; Gore, R. Working Conditions and Mental Health of Nursing Staff in Nursing Homes. *Issues Ment. Health Nurs.* **2016**, *37*, 485–492, doi:10.3109/01612840.2016.1162884.
16. Dill, J.; Duffy, M. Structural Racism And Black Women's Employment In The US Health Care Sector. *Health Aff. Proj. Hope* **2022**, *41*, 265–272, doi:10.1377/hlthaff.2021.01400.
